# Ensemble Classification Model With CFS-IGWO–Based Feature Selection for Cancer Detection Using Microarray Data

**DOI:** 10.1155/2024/4105224

**Published:** 2024-10-17

**Authors:** Pinakshi Panda, Sukant Kishoro Bisoy, Sandeep Kautish, Reyaz Ahmad, Asma Irshad, Nadeem Sarwar

**Affiliations:** ^1^Department of Computer Science & Engineering, C. V. Raman Global University, Bidyanagar, Mahura, Janla 752054, Bhubaneswar, Odisha, India; ^2^Apex Institute of Technology, Chandigarh University, Mohali, Punjab, India; ^3^School of General Education, Skyline University College, Sharjah, UAE; ^4^School of Biochemistry and Biotechnology, University of the Punjab, Lahore, Pakistan; ^5^Department of Computer Science, Bahria University Lahore Campus, Lahore 54600, Pakistan

**Keywords:** cancer, correlation feature selection (CFS), improved grey wolf optimizer (IGWO), microarray

## Abstract

Cancer is the top cause of death worldwide, and machine learning (ML) has made an indelible mark on the field of early cancer detection, thereby lowering the death toll. ML-based model for cancer diagnosis is done using two forms of data: gene expression data and microarray data. The data on gene expression levels includes many dimensions. When dealing with data with a high dimension, the efficiency of an ML-based model is decreased. Microarray data is distinguished by its high dimensionality with a greater number of features and a smaller sample size. In this work, two ensemble techniques are proposed using majority voting technique and weighted average technique. Correlation feature selection (CFS) is used for feature selection, and improved grey wolf optimizer (IGWO) is used for feature optimization. Support vector machines (SVMs), multilayer perceptron (MLP) classification, logistic regression (LR), decision tree (DT), adaptive boosting (AdaBoost) classifier, extreme learning machines (ELMs), and K-nearest neighbor (KNN) are used as classifiers. The results of each distinct base learner were then combined using weighted average and majority voting ensemble methods. Accuracy (ACC), specificity (SPE), sensitivity (SEN), precision (PRE), Matthews correlation coefficient (MCC), and F1-score (F1-S) are used to assess the performance. Our result shows that majority voting achieves better performance than the weighted average ensemble technique.

## 1. Introduction

Cancer has long been a leading cause of death worldwide, making it a significant global public health concern. Cancer is a broad term that encompasses several diseases resulting from the uncontrolled growth and spread of abnormal cells. It exerts a significant impact on the overall well-being of the general population and influences people from all age groups and backgrounds. It is a significant factor contributing to illness and mortality worldwide. Cancer mortality rates fluctuate both on a yearly basis and vary depending on location. According to the World Health Organization (WHO) and the National Cancer Institute, the number of cancer diagnoses in 2018 exceeded 18 million, resulting in over 10 million deaths attributed to inadequate diagnostic systems. The WHO estimates that in 2019, almost 9.6 million individuals succumbed to cancer on a global scale. It is estimated that approximately 10 million individuals globally will lose their lives to cancer in 2020 [1]. In 2021, the WHO reported that 9.9 million individuals died due to cancer. Approximately 20 million new cancer cases and 9.7 million deaths were recorded in 2022. Globally, cancer deaths vary by location. Due to modern medical care, faster diagnosis, and better drugs, wealthier countries have higher incidence and survival rates [2]. Cancer survival has improved with early detection and therapy [3]. The WHO, ACS, and other national cancer organizations seek to enhance cancer prevention, healthcare access, and public awareness [4]. Lifestyle changes, healthcare access, and medical research may impact global cancer rates. Public health and medical research prioritize cancer prevention and treatment [5]. By improving early detection, diagnosis, treatment, and patient outcomes, machine learning (ML) could significantly reduce cancer fatalities [6]. CT and mammography ML algorithms can detect cancer early. Early detection enhances treatment [7]. Delivering optimal pharmacological therapy to patients in a realistic timeframe may prevent diagnostic errors. ML predicts treatment outcomes using medical information, genetic data, and tumor features [8]. Through pharmaceutical research and development, ML may assist develop targeted cancer drugs. ML interprets cancer diagnosis data. Medical imaging and genomic data are crucial to ML-based cancer research [9]. Microarrays, gene expression profiling, and DNA sequencing improve cancer treatment, prevention, personalization, and efficacy [10]. Complexity, small sample size, and class imbalance make microarray data difficult for ML. Because microarray data is highly dimensional and has many genes or probes per sample or patient, typical ML methods may struggle with computational and cognitive resources [11]. Making diverse models with small samples is hard. Class imbalances can occur in cancer categorization when one class dominates. Researchers find cancer-diagnosis genes using reduced dimensionality, feature selection, and optimization [12].

### 1.1. Motivation

The inherent high degree of detail of microarray samples has the potential to impede the productivity of ML models. The presence of a significantly large number of dimensions poses many challenges; nonetheless, it is important to note that the act of lowering dimension may not always be a sufficient solution for addressing performance-related concerns. To improve ML model efficacy, the appropriate number of critical features must be identified without negatively impacting the model's efficacy.

### 1.2. Contribution

The contribution of the paper is summarized below:

• Finding relevant characteristics from the cancer dataset using correlation feature selection (CFS) technique and feature optimization using improved grey wolf optimizer (IGWO).
• Improving precision (PRE), a novel ensemble ML methodology using support vector machine (SVM), multilayer perceptron (MLP), logistic regression (LR), decision tree (DT), AdaBoost classifier, extreme learning machine (ELM), and K-nearest neighbor (KNN).
• Evaluate using weighted average and majority voting combining base learner results on cancer microarray datasets.
• Evaluate the performance of the model in terms of its accuracy (ACC), specificity (SPE), sensitivity (SEN), PRE, Matthews correlation coefficient (MCC), and F1-score (F1-S).

### 1.3. Paper Organization

The remainder of the paper is structured as follows: Based on the various approaches used to categorize cancer disorders, relevant scientific findings are presented in Section 2. The proposed technique for operation is outlined in Sections 3 and 4. The data analysis and related discussions are presented in Sections 5 and 6.  Section 7 discusses the proposed work with other studies. The work is concluded in Section 8 with a summary of potential future research.

## 2. Literature Survey

Entropy-based uncertainty measures in the neighborhood and ML algorithms including KNN, C4.5, and SVM helped Sun et al. identify colon, DLBCL, leukemia, lung, and SRBCT [13]. Ghoniem used Radiopaedia and LiTS datasets to develop a CNN, SegNet, UNet, and ABC liver cancer diagnostic model [14]. Shukla et al. developed an adaptive inertia weight teaching–learning model for breast, colon, DLBCL, leukemia, SRBCT, and lung cancers using SVM, ELM, and naive Bayes (NB) [15]. Global optimal feature selection and differential evolution discovered cancer data for Meenachi and Ramakrishnan. DT and ACO were applied to DLBCL, breast cancer, leukemia, SRBCT, and Gisette datasets [16]. Multifilter and adaptive chaotic multiobjective forest optimization algorithm (AC-MOFOA) hybrid solution considers FOA, ELM, MOO, and five filter techniques on nine datasets [17]. Yan et al.'s new feature selection model uses KNN and a wrapper technique on five datasets and is quite accurate [18]. The DT-based salp swarm optimization (DT-SWO) approach by Sarala, Chitraivel, and Raj merged ML and optimization [19]. Alomari et al. applied robust rMRMR, MGWO, and ML methods such as random forest (RF), elastic networks, and DT on nine datasets [20]. Balakrishnan, Dhanalakshmi, and Khaire upgraded salp swarm approach (iSSA) employing levy flight feature selection model and SVM classifier on six datasets [21]. Hameed et al. tested 12 datasets using SVM, NB, KNN, and RF and selected features and attributes with BPSO, genetic algorithm (GA), and CS [22]. SVM, NB, KNN, and RF were used to assess 12 datasets and a multiobjective graph theoretic-based approach model with CDNC to choose genes for microarray data categorization [23]. On six datasets, Aziz created a metaheuristic model using SVM, NB, ANN, cuckoo search, GA, and ABC [24]. Alromema, Syed, and Khan used SVM, KNN, neural networks (NNs), NB, DT, XGBoost, and LR for gene expression datasets [25]. Using NB, C4.5, GA, and ACO on 17 microarray expression datasets, Ke et al. suggested PIRC [26].  Table 1 shows a comprehensive review of several cancer illness classification algorithms.

## 3. Background Study

This section presents an in-depth analysis of diverse facts pertaining to ML, encompassing microarray data and cutting-edge methods.

### 3.1. Microarray Data

Microarray-based profiling is a highly efficient method for examining gene expression in cells or tissues across the entire genome. Nevertheless, when contrasting control and mutant samples, microarrays often identify a significant number of genes that exhibit differential expression. Two sorts of datasets can be employed in cancer diagnosis. Both the microarray dataset and the biopsy. The biopsy dataset does not encompass the patient's genetic information, but it does encompass the patient's laboratory test findings. The presence of genetic information has a significant impact on the diagnosis of cancer, so microarray data plays a crucial role in cancer diagnosis [2]. Microarray datasets have following characteristics.

#### 3.1.1. Small Sample Size

Compared to sample size, microarray data contains many patient attributes. Thus, the sample size may limit the classification. The application of classification techniques would reduce PRE and reduce the dimension to improve ACC.

#### 3.1.2. Class Variability

Class variance, another microarray data concept issue, occurs when some classes are major and others little. Class composition is uneven when the major class contains more properties and samples. This imbalance reduces PRE.

#### 3.1.3. Data Outliers

Some features in the microarray dataset can hinder performance and are labelled as such. Certain feature selection and extraction methods can eliminate these outliers.

#### 3.1.4. Data Redundancy

Microarray data is huge due to duplication. These characteristics may affect algorithmic prediction and categorization. Data preprocessing removes superfluous attributes before categorization or prediction.

## 4. Methodology

This section explains in details the dataset used for the experimental analysis with feature selection technique. In addition, ensemble techniques with different classifier used are explained below.

### 4.1. Dataset Description

There are five primary types of cancer databases [27], one of which is the leukemia cancer database, which comprises 72 distinct samples and 7129 distinct features that contain genetic information. In lung cancer, sample means the number of patients and 12533 numbers of genetic information of patients, prostate cancer 103 numbers samples and 341 numbers of attributes. In colon tumors 60 numbers of samples and 2000 numbers of attributes, and in breast cancer 32 numbers of samples and 570 numbers of attributes are taken and which is available athttps://csse.szu.edu.cn/staff/zhuzx/Dataset.html which is publicly accessible. Colon cancer and prostate cancer datasets are available in Kaggle and UCI websites. Additionally, a comprehensive summary of the dataset that was utilized in this investigation can be seen in Table 2.

### 4.2. CFS

The significant correlation between attributes suggests a strong linear dependence, hence indicating a comparable impact on the variable under study. It is possible to eliminate one of two tightly interconnected traits. The selection method (CFS) [21] is employed to determine the correlation between features within the sampled data.

Relationship coefficient was used to determine the degree to which the feature link is supported by statistical evidence. In light of this, let us formulate it as follows:

*M*_SC_ is the metric score and Dt is the dataset with *i* features.

(1) $$\begin{array}{c} M_{\text{SC}}=\frac{i\mu_{ic}}{\sqrt{i+i\left(i-1\right)\mu_{ii}}} \end{array}$$

where *μ*_*ic*_ is the average correlation of feature with target class c and *μ*_*ii*_ is the average of interfeature correlation. The correlation *μ*_*ab*_ between two features can be calculated as follows:

(2) $$\begin{array}{c} \mu_{ab}=\frac{\sum \left(a-\bar{a}\right)\left(\text{b}-\bar{b}\right)}{\sqrt{\left[\sum {\left(a-\bar{a}\right)}^{2}\right]}\left[\sqrt{\sum {\left(a-\bar{a}\right)}^{2}}\right]} \end{array}$$

where *a* and *b* are the values of the dependent and independent variables, respectively, where as $\;\bar{a}$ and $\bar{b}$ are the means of *a* and *b*.

### 4.3. IGWO

The metaheuristic optimization approach was inspired by the innate social structure and hunting strategies exhibited by grey wolves. The primary objective of this system is to effectively address intricate optimization challenges. The IGWO algorithm, a subfield within the realm of swarm intelligence, derives its inspiration for collective behaviour from the actions exhibited by animal populations [28]. Chaotic mappings have a beneficial effect on the rate at which grey wolf optimization (GWO) algorithms converge in optimization. Chaotic sequences possess traits such as nonlinearity, ergodicity, and the ability to avoid algorithms from getting stuck in local optima. Chaotic mapping has become extensively utilized in the past 10 years to enhance the optimization of dynamic and global search spaces for intelligent algorithms. There are more than 10 mappings, including the logistic mapping, piecewise-linear chaotic system mapping (pwlcm), singer mapping, and tent mapping [29]. The best answer is designated *μ* while producing IGWO, whereas the second and third best solutions are regarded as *Ω* and the third *ψ*. Every other potential option that could still exist is regarded as *€*.

(3) $$\begin{array}{c} \overset{⟶}{E}=\left|\overset{⟶}{E}.{\overset{⟶}{Z}}_{P}\left(t\right)-\overset{⟶}{Z}\;\left(t\right)\right| \end{array}$$

(4) $$\begin{array}{c} \overset{⟶}{Z}\left(i+1\right)={\overset{⟶}{Z}}_{P}\;\left(t\right)-\overset{⟶}{B}.\overset{⟶}{E} \end{array}$$

where *t* is the current iteration and $\overset{⟶}{B}$ and $\overset{⟶}{E}$ are the coefficient vectors and ${\overset{⟶}{Z}}_{p}$ is the position vector of prey (global solution).

The vectors $\overset{⟶}{B}$ and $\overset{⟶}{E}$ are calculated as follows:

(5) $$\begin{array}{c} \overset{⟶}{B}=2\overset{⟶}{\text{a}}.{\overset{⟶}{r}}_{1}-\overset{⟶}{\text{a}} \end{array}$$

(6) $$\begin{array}{c} \overset{⟶}{E}=2.{\overset{⟶}{r}}_{2} \end{array}$$

where $\overset{⟶}{\text{a}}$ linearly decreased from 2 to 0 and **r**_1_ and **r**_2_ are random vectors in [0,1].

Since the *μ*, *φ*, and *€* have a better idea of where prey is likely to be, they typically serve as the hunt's guides. Based on the top search agent, the other search agents should adjust their positions.

The modification to their agent position is as follows:

(7) $$\begin{array}{c} {\overset{⟶}{E}}_{\mu }=\left|{\overset{⟶}{E}}_{1}.{\overset{⟶}{Z}}_{\mu }-\overset{⟶}{Z}\right|,{\overset{⟶}{E}}_{\varphi }=\left|{\overset{⟶}{E}}_{2}.{\overset{⟶}{Z}}_{\varphi }-\overset{⟶}{Z}\right|,{\overset{⟶}{E}}_{\in }=\left|{\overset{⟶}{E}}_{3}.{\overset{⟶}{Z}}_{\in }-\overset{⟶}{Z}\right| \end{array}$$

(8) $$\begin{array}{c} {\overset{⟶}{Z}}_{1}={\overset{⟶}{Z}}_{\mu }-{\overset{⟶}{B}}_{1}.\left({\overset{⟶}{E}}_{\mu }\right),{\overset{⟶}{Z}}_{2}={\overset{⟶}{Z}}_{\varphi }-{\overset{⟶}{B}}_{2}.\left({\overset{⟶}{E}}_{\varphi }\right),{\overset{⟶}{Z}}_{3}={\overset{⟶}{Z}}_{\in }-{\overset{⟶}{B}}_{3}.\left({\overset{⟶}{E}}_{\in }\right) \end{array}$$

(9) $$\begin{array}{c} \overset{⟶}{Z}\left(t+1\right)=\frac{{\overset{⟶}{Z}}_{1}+{\overset{⟶}{Z}}_{2}+{\overset{⟶}{Z}}_{3}}{3} \end{array}$$

(10) $$\begin{array}{c} \overset{⟶}{E}\prime_{\mu }=\left|{\overset{⟶}{E}}_{1}.{\overset{⟶}{Z}}_{\mu }-\overset{⟶}{Z}\right|,\overset{⟶}{E}\prime_{\varphi }=\left|E_{2}.{\overset{⟶}{Z}}_{\varphi }-\overset{⟶}{Z}\right|,\overset{⟶}{E}\prime_{\in }=\left|{\overset{⟶}{E}}_{3}.{\overset{⟶}{Z}}_{\in }-\overset{⟶}{Z}\right| \end{array}$$

(11) $$\begin{array}{c} \overset{⟶}{Z}\prime_{1}={\overset{⟶}{Z}}_{\mu }-{\overset{⟶}{B}}_{1}.\left(\overset{⟶}{E}\prime_{\mu }\right),\overset{⟶}{Z}\prime_{2}={\overset{⟶}{Z}}_{\varphi }-{\overset{⟶}{B}}_{2}.\left(\overset{⟶}{E}\prime_{\varphi }\right),{\overset{⟶}{Z}}_{3}=\overset{⟶}{Z}\prime_{\in }-{\overset{⟶}{B}}_{3}.\left(\overset{⟶}{E}\prime_{\in }\right) \end{array}$$

(12) $$\begin{array}{c} {\overset{⟶}{Z}}^{\prime }\left(t+1\right)=\frac{\overset{⟶}{Z}\prime_{1}+\overset{⟶}{Z}\prime_{2}+\overset{⟶}{Z}\prime_{3}}{3} \end{array}$$

The IGWO optimization algorithm is an enhanced version of the original GWO method, designed to achieve improved performance in terms of convergence time, solution quality, and robustness across various optimization problem domains. The achievement of this objective is facilitated by the utilization of a computational technique known as the extended GWO algorithm.

### 4.4. DT

DT introduces a highly promising method for automating the majority of the data mining and predictive modelling process. They incorporate automated methods like overfitting and managing missing data. The models constructed by DTs can be readily perceived as a hierarchical structure of uncomplicated choices and offer cohesive answers with a high level of PRE [30, 31]. A DT, sometimes referred to as a classification tree, is a hierarchical structure that divides a dataset based on its attributes through recursive partitioning.

### 4.5. Linear Regression

It is a statistical technique employed to describe the correlation between a dependent variable and one or more independent variables. The relationship is postulated to be linear, indicating that every alteration in the independent variable(s) is linked to a commensurate alteration in the dependent variable. The objective of linear regression is to determine the optimal straight line that minimizes the discrepancy between the observed values and the values predicted by the model [32, 33].

### 4.6. AdaBoost

AdaBoost (adaptive boosting) is a well-known ML ensemble method often used for classification problems. A powerful ensemble classifier is built by combining the predictions of many less effective classifiers (typically DTs). AdaBoost uses the performance of the weak learners to change the weights, giving greater importance to the stronger ones [34].

### 4.7. SVM

SVM is a supervised classification and regression tool in the arena of ML. It is often used for classifying data. SVMs are well-known for their adaptability to high-dimensional data as well as their efficiency in locating a hyperplane that effectively splits data points into distinct groups. Finding a hyperplane that produces the highest difference across dataset classifications is the purpose of SVMs. The gap is the support vector distance from the hyperplane to the closest data points in each class. SVM attempts to obtain strong generalisation performance on unseen data by optimizing this margin [35].

### 4.8. MLP

MLP, is a distinct type of NN that comprises multiple layers and serves as a highly potent tool for predictive modelling. The structure comprises many layers, with each layer containing a specific number of connected neurons. These neurons get input by calculating the dot product between the neurons of the previous layer and the intermediate weight. In a multilayer architecture, the output of these neurons is passed on to the next layer of neurons, which is referred to as the hidden layer, and this process continues. The final layer is referred to as the output layer. This layer takes the weighted output of the previous hidden layer as its input. The result generated by the output layer after applying the activation function is referred to as the network's prediction for the provided tuples. MLP learns by iteratively processing the training tuples and evaluating the predictions against the known target values. The comparison output is commonly referred to as an error, which is subsequently utilized to adjust the weight of the network [36, 37].

### 4.9. ELM

For supervised learning tasks like classification and regression, utilize the ML method ELM. ELM is well-known for its computing efficiency and how easy it is to implement. For feedforward NNs with a single hidden layer, it was developed as an alternative to conventional NNs. In ELM, the feature vectors from the dataset are first fed into an input layer. In this setup, each feature is treated as a separate input node. In ELM, the number of hidden neurons or nodes is set beforehand, and there is just one hidden layer. Regression and classification may be performed in ELM's output layer. The output layer gives the continuous valued predictions for regression tasks directly. The output layer may use several activation functions for classification tasks [38].

### 4.10. KNNs

KNN is a type of supervised ML algorithm that stores every available example and then classifies new cases based on how similar they are to the stored cases. This approach is simple to implement since it does not require the building of the model or the fine-tuning of its parameters [39].

### 4.11. Voting Ensemble Classifier

The ML the majority voting classifier is used as an ensemble learning approach, for both classification and regression applications. It is a simple and powerful strategy for integrating the results of numerous models into a single forecast. When many models provide varying results and a group choice must be made based on those results, majority voting is an effective tool. Several different starting points are used in a majority voting ensemble. Classifiers and regressors like DTs, SVMs, and LR are only examples of what might serve as these foundational models. For majority voting to work, it needs a wide variety of these starting points. On the same dataset, we utilize each base model to generate predictions. A vote decides the designation of each group's category. The ensemble's final forecast is the class label that wins the most votes from the base models [40].

### 4.12. Averaging Ensemble Classifier

This ensemble classifier referred to as an averaging or mean ensemble is a method of combining the predictions of base models by calculating their average [41]. An averaging ensemble, often referred to as model averaging or ensemble averaging, is a technique used in ML where predictions from multiple models are combined to make a final prediction. This approach is based on the principle that combining the predictions of multiple models can often lead to better performance than any single model alone. In averaging ensemble, the predictions from each individual model are combined using some form of averaging. The simplest form of averaging is arithmetic mean, where the predictions from all models are added together and then divided by the number of models. However, other forms of averaging, such as weighted averaging, can also be used, where each model's prediction is given a specific weight based on its performance or reliability.

## 5. Proposed Method

In order to enhance the performance of proposed model, the ensemble technique employs the aggregation of forecasts generated by multiple models. Voting and averaging are ensemble techniques that are quite straightforward to comprehend and use. Both methods are utilized in the tasks of classification and regression. Both methodologies commence by utilizing a set of training data and employing classification or regression techniques. Additionally, both methodologies involve the utilization of the same training dataset and follow a consistent strategy to divide the training dataset into multiple base models.

**Figure 1:**
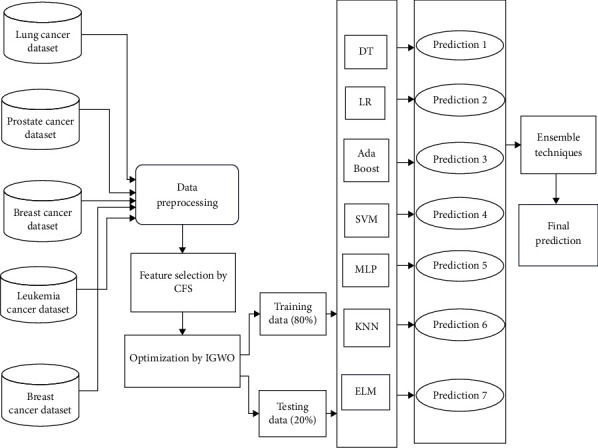
Flow chart of the proposed model.

The suggested model employs the CFS technique for feature selection and the IGWO technique for optimization. An ensemble method is offered as a means to enhance the ACC of projected outcomes. Various classifiers are utilized to classify the curated and optimized dataset. The cancer dataset undergoes an initial preprocessing phase, after which feature extraction techniques are applied. Subsequently, optimization algorithms are employed. The dataset is subsequently partitioned into training and testing using an 80:20 ratios. The flow of the proposed work is demonstrated in Figure 1.  Algorithm 1 under consideration is comprehensively elucidated in the provided pseudocode.

Initial predictions are processed using base learners including LR, KNN, SVM, DT, ELM, MLP, and AdaBoost. The final prognosis is generated by employing ensemble techniques to the initial prediction.

### 5.1. Workflow

To eliminate noisy data, the dataset is initially examined for normalization. A microarray dataset measures the gene expression levels for thousands of genes simultaneously. Normalization is a process that eliminates technical disparities between samples caused by factors including variations in RNA quality, labelling efficiency, and hybridization settings.

In the present research, we employed the standard scalar normalization technique to normalize microarray datasets. This method involves scaling the characteristics so that they have a mean of 0 and a standard deviation of 1. This normalization technique is aimed at standardizing different features by bringing them to a similar scale. The process involves computing the mean and standard deviation of each feature and then normalizing each feature accordingly. As a result of this normalization process, each feature will have a mean of zero and a standard deviation within a specific range. This normalization is applied across the entire dataset. The normalization procedure in microarray datasets involves normalizing gene expression values across samples, with each gene's expression values being normalized independently.

After normalization of dataset, the CFS algorithm is applied to the normalized data to determine the optimal feature. IGWO is subsequently implemented as an optimizer. The operational aspects of the proposed paradigm are illustrated in Figure 1.

The proposed procedure works as follows.

• Step 1: consider the dataset for normalization by using standard scalar normalization process.
• Step 2: choose relevant attributes using the CFS algorithm.
• Step 3: optimize the featured dataset with IGWO.
• Step 4: make an 80:20 dataset split.
• Step 5: based on training data, train seven models (LR, KNN, SVM, DT, ELM, MLP, and AdaBoost).
• Step 6: select three most accurate classifiers.
• Step 7: obtain the final forecast estimate using ensemble methods.
• Step 8: evaluate performance.

## 6. Results and Discussion

The suggested model is developed using Python 3.11 on Ubuntu 20.04 with 32 GB of RAM, an Intel Core i7 CPU and a 1 TB SSD. The empirical analysis has been done in three different approaches. Approach 1 shows the performance of all considered classifiers with CFS feature selection algorithm. Approach 2 shows the performance of all considered classifiers with CFS and IGWO optimizer. Approach 3 shows the performance of the proposed ensemble classifier with three best classifiers from Approach 2 based on ACC. Initially, classification methods LR, KNN, SVM, DT, ELM, MLP, and AdaBoost are considered. Then, two ensemble learning methods are proposed to improve the performance of the models. For evaluation, many performance metrics are considered. True negatives are Trn and false negatives are Fln and true positive and false positive are Trp and Flp. The performance in all of the above said approaches is based on six different parameters including ACC, PRE, SEN, SPE, F1-S, and MCC. Equations (13)–(18) show the performance evaluation parameters:

(13) $$\begin{array}{c} \text{ACC}=\frac{\text{Trp}+\text{Trn}}{\text{Trp}+\text{Trn}+\text{Flp}+\text{Fln}} \end{array}$$

(14) $$\begin{array}{c} \text{SEN}=\frac{\text{Trp}}{\text{Trp}+\text{Fln}} \end{array}$$

(15) $$\begin{array}{c} \text{SPE}=\frac{\text{Trn}}{\text{Trn}+\text{Flp}} \end{array}$$

(16) $$\begin{array}{c} \text{PRE}=\frac{\text{Trp}}{\text{Trp}+\text{Flp}} \end{array}$$

(17) $$\begin{array}{c} {\text{F}}_{1\text{s}}=\frac{2\times \text{Pre}\times \text{Sen}}{\text{Pre}+\text{Sen}} \end{array}$$

(18) $$\begin{array}{c} \text{MCC}=\frac{\left(\text{Trp}+\text{Trn}\right)-\left(\text{Flp}+\text{Fln}\right)}{\sqrt{\left(\text{Trp}+\text{Flp}\right)\left(\text{Trp}+\text{Fln}\right)\left(\text{Trn}+\text{Fln}\right)\left(\text{Trn}+\text{Flp}\right)}} \end{array}$$

where Trp is the true positive, Trn is the true negative, Flp is the false positive, and Fln is the false negative.

Implemented CFS as feature reduction and with the IGWO algorithm for featured dataset was optimized. The dimensionality of the different cancers is shown in Table 3 after CFS and IGWO technique.

Table 4 lists the feature selection quantities by level. Results of voting and averaging ensemble methods were analyzed. These hybrid models CFS + SVM, CFS + MLP, CFS + ELM, CFS + AdaBoost, CFS + DT, CFS + KNN, and CFS + LR perform with diverse cancer gene expression datasets. The performance of many hybrid models is compared in Table 4 using five different gene expression datasets (*D*_1_, *D*_2_, *D*_3_, *D*_4_, and *D*_5_).

Table 4 shows the performance of different classifiers using CFS on the different cancer dataset.

The ACC of the CFS-ELM model in the lung cancer dataset is 80.17%, which is higher than the ACC of other models. The PRE, MCC, F1-S, SEN, and SPE are 81.82%, 83.72%, 82.76%, 81.33%, and 80.41%, respectively.

The ACC of the colon tumor dataset in CFS-AdaBoost is 83.87%. The PRE, MCC, F1-S, SEN, and SPE values are 83.78%, 84.71%, 86.81%, 86.54%, and 80.23%, respectively.

On the prostate dataset, the CFS + DT hybrid model has the highest ACC of 79.17%. The PRE, MCC, F1-S, SEN, and SPE scores are 81.43%, 86.05%, 83.15%, 84.86%, and 75.97%, respectively.

CFS + AdaBoost surpasses other methods in the breast gene expression dataset, with 80.72% ACC. The PRE, MCC, F1-S, SEN, and SPE scores are 84.56%, 83.72%, 81.82%, 82.95%, and 77.51%, respectively.

The CFS + MLP performs well on the leukemia gene expression dataset, with an ACC of 78.34%. The PRE, MCC, F1-S, SEN, and SPE scores are 80.11%, 82.13%, 81.45%, 82.89%, and 85.78%, respectively.

The feature set obtained by the CFS algorithm is optimized by the IGWO algorithm to improve hybrid model functionality. CFS + IGWO + DT, LR, MLP, KNN, SVM, ELM, and AdaBoost hybrid models will be tested on cancer gene expression datasets. Tables 5, 6, 7, 8, 9, 10, and 11 compared each model performance on different datasets.

CFS-IGWO-DT performance in five cancer datasets is shown in Table 5. The Lung cancer has the highest PRE (94.8) and ACC (94.3). CFS-IGWO-LR's performance on five cancer datasets is in Table 6. The Breast cancer was the most precision at 93.5 and lung cancer was the least at 94.7.  Table 7 shows CFS-IGWO-MLP's ACC 95.7 and PRE 89.9 for five cancer datasets, with lung cancer performing best. CFS-IGWO-KNN performance on five cancer datasets is in Table 8. Breast cancer is 90.7, whereas lung cancer is 95.7. CFS-IGWO-SVM performance on five cancer datasets is in Table 9. These datasets include 97.8% lung cancer ACC and 94.3% colon tumor PRE. The CFS-IGWO-ELM algorithm's performance on five cancer datasets is shown in Table 10. The breast cancer dataset has 97.2 ACC and 96.5 PRE. On five cancer datasets, Table 11 illustrates CFS-IGWO-AdaBoost performance having the highest ACC with 97.5 for leukemia cancer and 97.9 for lung cancer.

**Figure 2:**
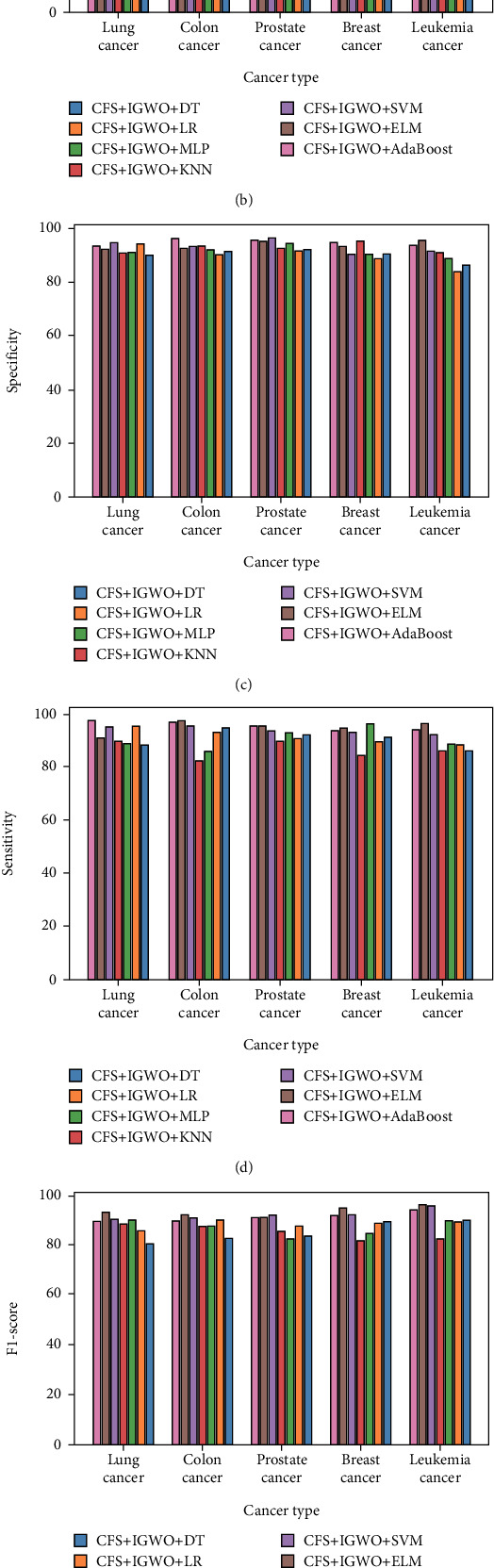
Performance measure of (a) accuracy, (b) precision, (c) specificity, (d) sensitivity, (e) F1-score, and (f) MCC.

Figure 2 shows the performance measure of ACC, PRE, SPE, SEN, F1-S, and MCC of five different datasets.

Figure 2(a) shows model ACC compared to datasets. CFS + IGWO + DT, LR, MLP, KNN, SVM, ELM, and AdaBoost are lung cancer models. The ACC of these models is 94.3, 93.5, 95.7, 97.8, 96.7, and 97.9. CFS + IGWO + AdaBoost have the highest ACC of 97.9. For colon cancer analysis, CFS + IGWO + DT, LR, MLP, KNN, SVM, ELM, and AdaBoost are used. Model ACC is 92.2, 92.9, 90.9, 94.3, 96.8, 95.7, and 96.8. With 96.8% ACC, the CFS + IGWO + SVM and AdaBoost model is the most accurate. Models for prostate cancer include CFS + IGWO + DT, LR, MLP, KNN, SVM, ELM, and AdaBoost. The ACC of these models is 91.3, 90.5, 93.8, 90.7, 96.4, 94.6, and 94.6. The CFS + IGWO + SVM model has the highest ACC of 96.4. The breast cancer models are CFS + IGWO + DT, LR, MLP, KNN, SVM, ELM, and AdaBoost. These models have 89.9, 94.7, 94.5, 93.7, 95.6, 97.2, and 96.8 ACC. The CFS + IGWO + ELM model has the highest ACC of 97.2. The models CFS + IGWO + DT, LR, MLP, KNN, SVM, ELM, and AdaBoost show leukemia cancer. The models' ACC rates are 88.1, 89.9, 97.9, 90.6, 97.3, 94.3, and 97.5. With 97.9% ACC, the CFS + IGWO + MLP model is the most accurate.

Figure 2(b) presented a comparison of the PRE of different models in relation to their respective datasets. The models used to research lung cancer are CFS + IGWO + DT, LR, MLP, KNN, SVM, ELM, and AdaBoost. The models had PREs of 94.3, 93.2, 89.9, 89.4, 93.6, 90.4, and 92.8. The CFS + IGWO + DT model has the highest PRE rate, 94.3%. The analysis of colon cancer uses CFS + IGWO + DT, LR, MLP, KNN, SVM, ELM, and AdaBoost models. The models' PREs are 93.1, 89.6, 85.8, 87.3, 94.3, 91.5, and 95.6. The most precise model was the CFS + IGWO + AdaBoost model, with 95.6% PRE. For prostate cancer, CFS + IGWO + DT, LR, MLP, KNN, SVM, ELM, and AdaBoost are used. The models' PRE values are 92.2, 88.8, 87.9, 86.5, 92.2, 94.5, and 97.5. This model's PRE is 97.5, the highest among the collection. For breast cancer, CFS + IGWO + DT, LR, MLP, KNN, SVM, ELM, and AdaBoost are used. The models had PREs of 90.2, 87.3, 86.9, 90.7, 91.5, 96.5, and 93.5. The maximum PRE is 96.5 for the CFS + IGWO + ELM model. Several models represent leukemia cancer, including CFS + IGWO + DT, LR, MLP, KNN, SVM, ELM, and AdaBoost. Percentages of 86.1, 88.4, 88.2, 88.4, 90.6, 93.4, and 94.3 show these models' PRE. The highest PRE achieved is 94.3% for the CFS + IGWO + AdaBoost model.

Figure 2(c) presents a comparison of the SPE of different models in relation to their respective datasets. Lung cancer models include CFS + IGWO + DT, LR, MLP, KNN, SVM, ELM, and AdaBoost. These models feature specificities of 89.9%, 94.2%, 90.9%, 90.8%, 94.5%, 91.8%, and 93.5%. The CFS + IGWO + SVM model showed the highest SPE in the values analyzed, 94.5%. Colon cancer was analyzed using CFS + IGWO + LR, DT, MLP, KNN, SVM, ELM, and AdaBoost. SPE values for models include 91.2, 90.3, 92.3, 93.4, 92.2, and 96.4. The CFS + IGWO + AdaBoost model got the highest SPE, 96.4%. CFS + IGWO + DT, LR, MLP, KNN, SVM, ELM, and AdaBoost prostate cancer models were utilized. Model SPE values are 92.3%, 91.3%, 94.5%, 92.5%, 96.7%, 95.4%, and 95.6%. The highest SPE model is CFS + IGWO + SVM at 96.7%. The research used CFS + IGWO + DT, LR, MLP, KNN, SVM, ELM, and AdaBoost breast cancer models. The models have specificities of 90.2, 88.6, 90.4, 95.3, 90.6, 93.6, and 94.6. The CFS + IGWO + KNN model has the highest SPE of 95.3. Leukemia cancer models include CFS + IGWO + DT, LR, MLP, KNN, SVM, ELM, and AdaBoost. Percentages of 86.2, 83.6, 88.7, 91.1, 91.5, 95.6, and 93.8 indicate the SPE of these models. Among all models evaluated, CFS + IGWO + ELM has the highest SPE of 95.6%.

Figure 2(d) compares the SEN of different models to their respective datasets. The models CFS + IGWO + DT, LR, MLP, KNN, SVM, ELM, and AdaBoost have all been used to study lung cancer. The models have SEN values of 88.3, 95.4, 88.9, 94.9, 90.8, and 97.4. The CFS + IGWO + AdaBoost model has the highest SEN, at 97.4%. Multiple models, such as CFS + IGWO + DT, LR, MLP, KNN, SVM, ELM, and AdaBoost, are utilized to investigate colon cancer. The CFS + IGWO + ELM model achieved a SEN rate of 97.6%, which was the greatest among all the models. The SEN rates for the other models were 94.8%, 93.2%, 85.9%, 82.5%, 95.7%, 97.6%, and 96.8%. Prostate cancer models encompass CFS + IGWO + DT, LR, MLP, KNN, SVM, ELM, and AdaBoost. The SEN values of these models are 92.1, 90.4, 92.8, 89.7, 93.6, 95.4, and 95.6. The highest SEN is 95.6 for the CFS + IGWO + AdaBoost model. For breast cancer, CFS + IGWO + DT, LR, MLP, KNN, SVM, ELM, and AdaBoost are applied. The CFS + IGWO + AdaBoost model has the maximum SEN, 96.8. Leukemia cancer is depicted using CFS + IGWO + DT, LR, MLP, KNN, SVM, ELM, and AdaBoost models. The CFS + IGWO + ELM model has the maximum (96.5%) SEN.

Figure 2(e) presents a comparison of the F1-Ss of different models in relation to their respective datasets. Lung cancer was studied using CFS + IGWO + DT, LR, MLP, KNN, SVM, ELM, and AdaBoost models. The model F1-S are 80.1, 85.8, 89.9, 88.4, 89.9, 92.7, and 88.9. The CFS + IGWO + ELM model obtained the highest F1-S in the study, 92.7%. Colon cancer is explored utilizing CFS + IGWO + DT, LR, MLP, KNN, SVM, ELM, and AdaBoost models. Of the models studied, the CFS + IGWO + ELM model had the highest F1-S rate of 91.8%, with 82.2, 89.7, 86.9, 87.3, 90.3, 91.8, and 89.6. Prostate cancer models include CFS + IGWO + DT, LR, MLP, KNN, SVM, ELM, and AdaBoost. F1-Ss for these models are 83.1, 87.4, 82.2, 85.3, 91.7, 90.7, and 90.4. CFS + IGWO + SVM's F1-S is 91.7, the highest of the values. For breast cancer, CFS + IGWO + DT, LR, MLP, KNN, SVM, ELM, and AdaBoost are used. The models have F1-Ss of 89.1, 88.5, 84.4, 81.2, 91.8, 94.6, and 91.7. The greatest F1-S is 94.6 for the CFS + IGWO + ELM model. Leukemia cancer is depicted using CFS + IGWO + DT, LR, MLP, KNN, SVM, ELM, and AdaBoost models. The CFS + IGWO + ELM model has the greatest F1-S 95.7%.

Figure 2(f) presents a comparison of the MCC of different models in relation to their respective datasets. Lung cancer studies used the models CFS + IGWO + DT, LR, MLP, KNN, SVM, ELM, and AdaBoost. Model MCC values are 90.3, 91.8, 90.2, 90.5, 88.9, 90.7, and 94.8. CFS + IGWO + AdaBoost had the highest MCC rate, 94.8%. For colon cancer research, CFS + IGWO + ELM, DT, LR, MLP, KNN, SVM, and AdaBoost are used. CFS + IGWO + MLP had the greatest MCC rate (92.9%) of the models examined. Prostate cancer models include CFS + IGWO + ELM, LR, MLP, KNN, SVM, and AdaBoost. The following are the models' MCC values: 81.2, 89.5, 90.8, 88.2, 87.4, 88.6, and 93.7. CFS + IGWO + AdaBoost has the highest MCC, 93.7. Breast cancer models include LR, MLP, KNN, SVM, ELM, and AdaBoost. The following are the values of MCC model: 81.4, 87.8, 94.6, 84.4, 90.1, 93.5, and 91.5. The highest MCC is 94.6 for CFS + IGWO + MLP. Leukemia cancer is shown using CFS + IGWO + DT, LR, MLP, KNN, SVM, ELM, and AdaBoost. A few models had MCC percentages of 83.3, 85.3, 86.1, 85.3, 91.7, 92.9, and 89.9. At 92.9%, CFS + IGWO + ELM had the greatest MCC. The averaging and voting collaborative approaches were used to improve the efficacy of ML classifiers. Ensemble approaches were used to improve the PRE of ML classifiers after calculating the ACC, PRE, SPE, SEN, F-measure, and MCC for several models.

### 6.1. Analysis of Ensemble Classifier

Table 12 shows lung cancer dataset results from averaging and voting ensemble classifiers. Averaging is less accurate than voting's 99.1%.  Table 13 shows colon tumor dataset results for averaging and voting ensemble classifiers. Better than averaging, voting classifier ACC was 99.1%.  Table 14 shows prostate cancer dataset averaging and voting ensemble classifier results. The voting classifier ACC was 99.3%. Averaging and voting ensemble classifiers were applied to breast cancer dataset in Table 15. Voting outperformed averaging with 99.2% ACC.  Table 16 shows averaging and voting ensemble classifier results for leukemia cancer. As compared to averaging technique, voting classifier has higher ACC of 98.7%.

**Figure 3:**
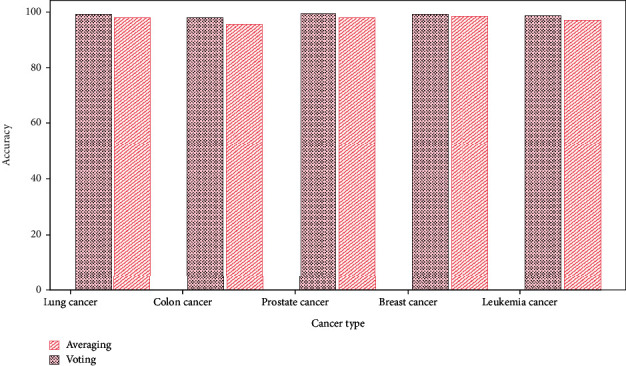
Accuracy comparison of averaging and voting ensemble classifier.

Figure 3 shows the averaging and voting ensemble classifier ACC on their datasets. The average lung cancer ensemble ACC is 97.9% and voting ACC is 99.1%. Averaging and voting ACC for colon cancer detection are 95.6% and 97.8%. In prostate cancer detection, averaging and voting ACC are 97.8% and 99.3%. Breast cancer ACC is 98.2%–99.2% and leukemia cancer is 97.1%–98.7%.

**Figure 4:**
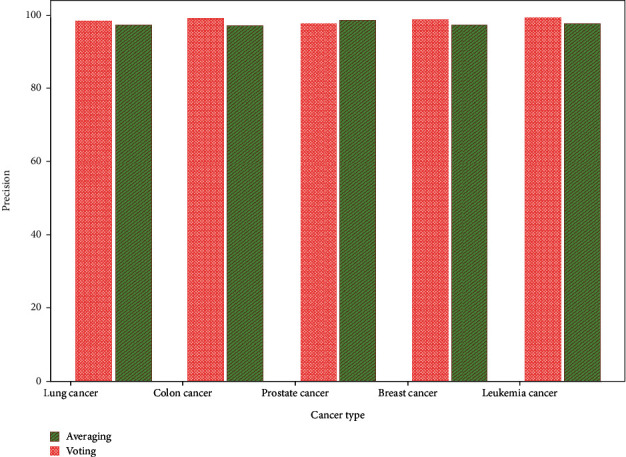
Precision comparison of averaging and voting ensemble classifier.

Averaging and voting ensemble classifier PRE are compared in Figure 4. While voting is 98.3% precise, lung cancer ensembles average 96.7%. Voting and averaging identify colon cancer with 96.7% and 98.7% ACC. Average prostate cancer detection PRE is 98.4%, voting 97.6%. Breast cancer PRE is 97.2%–98.4%. The ACC of leukemia cancer is 97.4%–99.1%.

**Figure 5:**
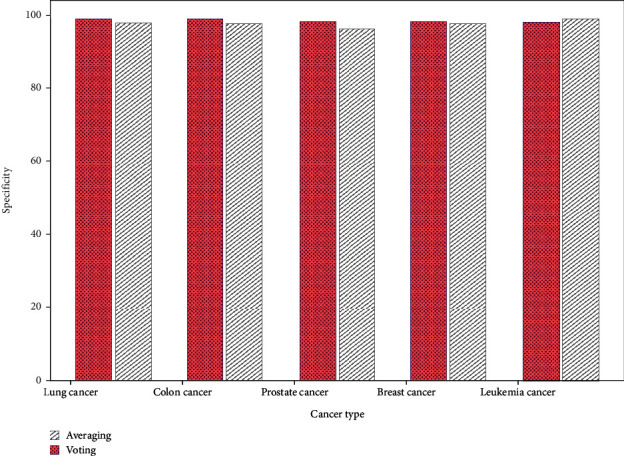
Specificity comparison of averaging and voting ensemble classifier.

The SPE of averaging and voting ensemble classifiers are compared to their datasets in Figure 5. Voting PRE is 99.2% and PRE is 98.1% for lung cancer. In colon cancer detection, the averaging and voting techniques have 97.9% and 99.1% SPE. Both averaging and voting approaches identify prostate cancer with 96.4% and 98.4% SPE. Breast cancer had 97.8% and 98.2% SPE. Leukemia cancer SPE is 99.1% and 98.2%.

**Figure 6:**
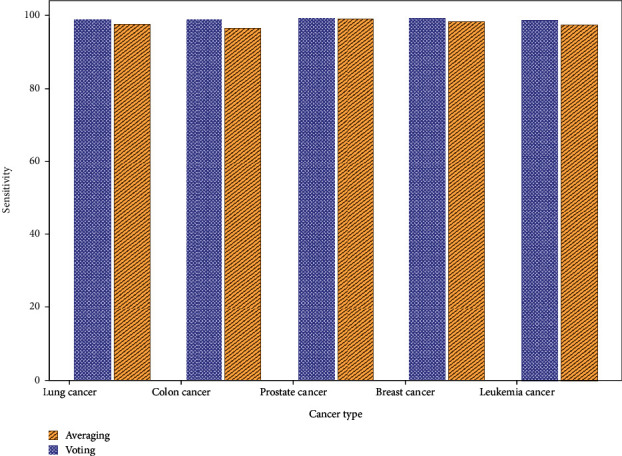
Sensitivity comparison of averaging and voting ensemble classifier.

SEN of averaging and voting ensemble classifiers to their datasets is shown in Figure 6. For lung cancer, the ensemble has 97.3% and 98.8% vote SEN. Averaging and voting identify colon cancer with 96.4% and 98.9% SEN. Voting and averaging detect prostate cancer with 98.9% and 99.2% SEN. Breast cancer SEN is 98.2%–99.1%. SPE for leukemia is 97.3%–98.6%.

**Figure 7:**
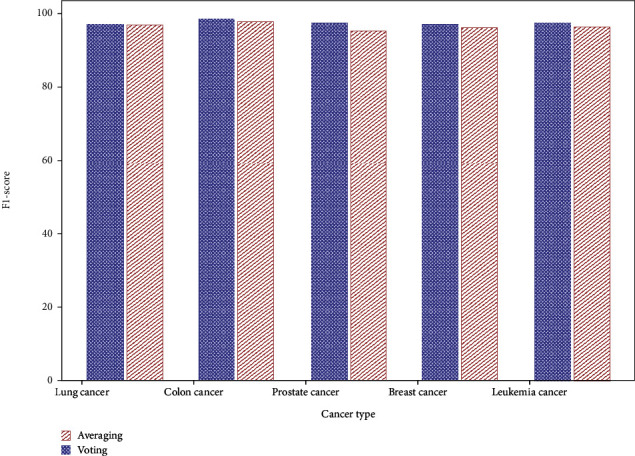
F1-score comparison of averaging and voting ensemble classifier.

Figure 7 presents a comparison of the F1-S of averaging and voting ensemble classifier to their respective datasets.

The lung cancer ensemble averages 96.8% F1-S, although voting is 97.2%. Both approaches identify colon cancer with F1-Ss of 97.9% and 98.7%. Prostate cancer detection F1-Ss by averaging and voting are 95.4% and 97.5%. Breast cancer F1-Ss are 96.2% and 97.1%. Leukemia F1-Ss are 96.4% and 97.5%.

**Figure 8:**
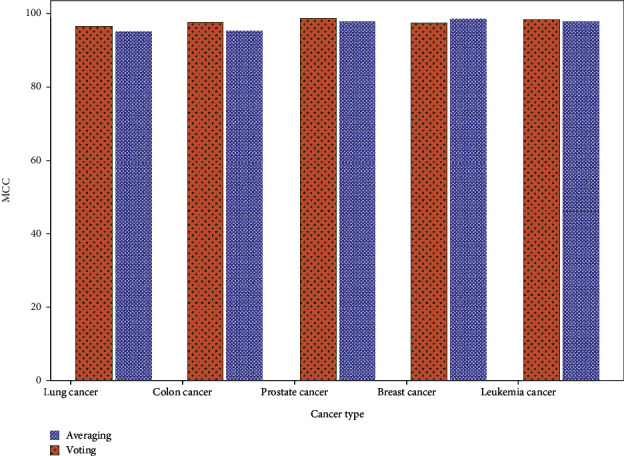
MCC comparison of averaging and voting ensemble classifier.

Figure 8 presents a comparison of the MCC of averaging and voting ensemble classifier to their respective datasets. The lung cancer ensemble averages 95.3% MCC, although voting is 96.4%. Colon cancer detection using averaging and voting had 95.2% and 97.4% MCC scores. Averaging and voting MCC prostate cancer detection rates are 97.7% and 98.6%. MCC values for breast cancer are 98.1% and 97.2%. Leukemia uses 97.8% and 98.2% MCC. Ensemble classifier findings favour voting over averaging. Compared to averaging, voting classifiers detect lung cancer 99.1% better. Breast, prostate, colon, and leukemia had ACC rates of 97.8%, 99.3%, 99.2%, and 98.7%. The averaging classifier is less accurate than majority voting for considered datasets.

The performance of averaging and voting ensemble classifier models is assessed using their respective datasets and presented in Table 17. The tables were generated by ensemble voting, incorporating data from lung, colon, prostate, breast, and leukemia cancers. Surpassing multiple categorization methods, it achieves an ACC of 99.1, 97.8, 99.3, 99.2, and 98.7.

## 7. Critical Analysis

We compare several earlier works of literature with the proposed technique stated above, in which we acquire the best results in voting ensemble classifier, using ACC as the criteria.  Table 18 displays an extensive comparison of the suggested study with the body of current research. According to the findings of the analysis, it is evident that the proposed work is superior to [13, 15] The ACC is 98.8% in the case of lung cancer, 83.8% and 98.3% in the case of colon cancer, and 92.9% and 98.9% in the case of leukemia cancer, respectively. In [17], prostate cancer has an ACC of 97.8%, and breast cancer has an ACC of 89.8%. The ACC of our proposed work is 99.1% for lung cancer, 97.8% for colon cancer, 99.3% for prostate cancer, 99.2% for breast cancer, and 98.7% for leukemia cancer.

## 8. Conclusion and Future Work

Artificial intelligence (AI) and related technologies, including ML, evolutionary learning, and deep learning, proved to be useful in the field of early cancer detection. In this work, we have proposed two ensembles of classifiers based on five datasets that use majority voting and weighted averages to predict cancer diseases. ACC, SEN, SPE, PRE, and the F1-S are some of the parameters used to evaluate the performance of the above models. The proposed approach incorporates the adoption of an ensemble technique known as majority voting, which strives to boost the performance of individual base learners. The voting method outperforms weighted average ensemble techniques on an array of cancer microarray datasets, with an ACC rate of 99.1%. In the future, a variety of cancer gene expression datasets can be utilized to evaluate the model's ACC. Furthermore, research involving multiple classes of data can be considered to do the analysis.

## Figures and Tables

**Algorithm 1:**
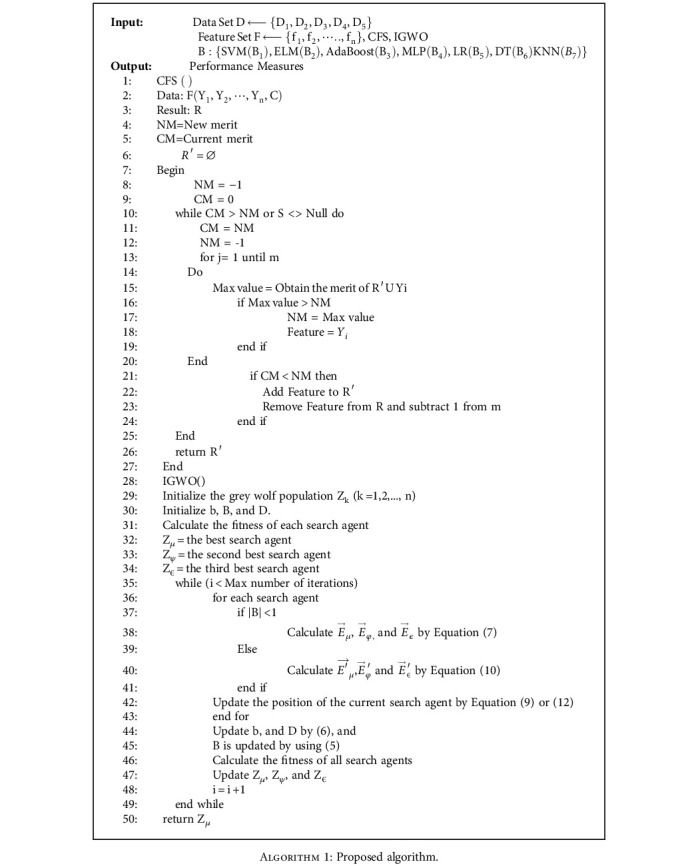
Proposed algorithm.

**Table 1:** Comparison of literature-based cancer disease classification technique.

**Ref**	**Techniques employed**	**Datasets employed**	**Findings**
[13]	KNN, C4.5, SVM	Colon	Accuracy: 83.8%
		DLBCL	Accuracy: 92.7%
		Leukemia	Accuracy: 92.9%
		Lung	Accuracy: 98.8%
		SRBCT	Accuracy: 93.6%

[14]	CNN along with SegNet and UNet, ABC	Radiopaedia datasets	Accuracy: 99.3%
			F1-score: 99.0%
			Specificity: 99.0%
		LiTS datasets	Accuracy: 99.5%
			F1-score: 98.0%
			Specificity: 99.0%

[15]	SVM, ELM, and NB	Breast cancer	Accuracy: 89.59%
		Colon cancer	Accuracy: 98.03%
		DLBCL	Accuracy: 99.89%
		Leukemia	Accuracy: 98.99%
		Lung cancer	Accuracy: 98.83%

[16]	DT and ACO	DLBCL	Accuracy: 92.65%
			Specificity: 98.4%
			Precision: 95.8%
			Recall: 95.4%
			F-measure: 95.1%
		Breast cancer	Accuracy: 71.88%
			Specificity: 91.1%
			Precision: 59.3%
			Recall: 72.1%
			F-measure: 64.3%
		Leukemia	Accuracy: 85.29%
			Specificity: 92.8%
			Precision: 89.6%
			Recall: 85.8%
			F-measure: 85.6%
		SRBCT	Accuracy: 81.58%
			Specificity: 93.1%
			Precision: 82.5%
			Recall: 82.1%
			F-measure: 81.7%
		Gisette	Accuracy: 87.41%
			Specificity: 87.3%
			Precision: 87.5%
			Recall: 87.3%
			F-measure: 87.4%

[17]	FOA, ELM, MOO	SRBCT	Accuracy: 90.72%
		Tumors_9	Accuracy: 84.41%
		Leukaemia_3	Accuracy: 97.66%
		Colon_prostate	Accuracy: 97.89%
		Lung	Accuracy: 93.97%
		GCM	Accuracy: 78.03%
		Breast	Accuracy: 86.53%
		Rsctc_5	Accuracy: 72.38%
		Rsctc_6	Accuracy: 82.69%

[18]	KNN, FS_SSA, PSO, and GA	ALL-AML-4	Accuracy: 94.23%
		Colon tumor	Accuracy: 82.09%
		Lymphoma	Accuracy: 88.57%
		MLL	Accuracy: 86.19%
		SRBCT	Accuracy: 76.74%

[19]	DT, SVM, NB, KSVM, and SWO	DLBCL	Accuracy: 95%
		Leukemia	Accuracy: 97%
		Lung cancer	Accuracy: 94%
		Colon	Accuracy: 98%

[20]	MRMR-MGWO, LASSO, RF, EN, and DT	Colon tumor	Accuracy: 94.14%
			Precision: 95.33%
			Recall: 91.97%
			F1-score: 95.46%
			MCC: 86.39
		CNS	Accuracy: 100%
			Precision: 100%
			Recall: 100%
			F1-score: 100%
			MCC: 100%
		AII-AML	Accuracy: 100%
			Precision: 100%
			Recall: 100%
			F1-score: 100%
			MCC: 100%
		Ovarian cancer	Accuracy: 100%
			Precision: 100%
			Recall: 100%
			F1-score: 100%
			MCC: 100%
		Lung cancer	Accuracy: 97.52%
			Precision: 94.45%
			Recall: 98.82%
			F1-score: 95.79%
			MCC: 92.0%
		AII-AML-3C	Accuracy: 99.86%
			Precision: 99.82%
			Recall: 99.94%
			F1-score: 99.77%
			MCC: 97%
		AII-AML-4C	Accuracy: 98.84%
			Precision: 99.11%
			Recall: 99.62%
			F1-score: 98.63%
			MCC: 93%
		MLL	Accuracy: 99.90%
			Precision: 99.89%
			Recall: 99.95%
			F1-score: 99.9%
			MCC: 98.0%

[21]	SSA, levy flight, SVM	OSCC	Accuracy: 85.7%
			F1-score: 85.7%
			Recall: 90%
			Precision: 90.0%
		Ovarian cancer	Accuracy: 83.33%
			F1-score: 84.3%
			Recall: 89.5%
			Precision: 88.0%
		Breast cancer	Accuracy: 50%
			F1-score: 66.6%
			Recall: 100%
			Precision: 50%
		CNS	Accuracy: 66.6%
			F1-score: 58.8%
			Recall: 45.4%
			Precision: 83.3%
		Colon cancer	Accuracy: 86.9%
			F1-score: 88.0%
			Recall: 100%
			Precision: 78.5%
		Leukemia	Accuracy: 85.7%
			F1-score: 87.5%
			Recall: 100%
			Precision: 100%

[22]	BPSO, GA, CS, KNN, SVM, NB, RF	Brain	Accuracy: 97.62%
		Breast	Accuracy: 86.60%
		CNS	Accuracy: 80.00%
		Colon	Accuracy: 93.55%
		Leukemia	Accuracy: 100%
		Lung	Accuracy: 97.54%
		Lymphoma	Accuracy: 100%
		Ovarian	Accuracy: 100%
		Prostate	Accuracy: 96.08%
		SRBCT	Accuracy: 100%
		TCGA	Accuracy: 100%

[23]	CDNC	Colon	Accuracy: 88.73%
		Leukemia	Accuracy: 90.18%
		SRBCT	Accuracy: 82.82%
		Prostate tumor	Accuracy: 82.91%
		Lung cancer	Accuracy: 91.76%

[24]	SVM, NB, and ANN along with CS, GA, and ABC	Colon cancer	Accuracy: 93.01%
		Acute leukemia	Accuracy: 93.35%
		Prostate tumor	Accuracy: 89.14%
		High-grade glioma	Accuracy: 90.32%
		Lung cancer II	Accuracy: 87.71%
		Leukemia_2	Accuracy: 93.67%

[25]	SVM, KNN, NN, NB, DT, XGBoost, LR	Gene expression dataset	Accuracy: 97.6%
			F1-score: 97.4%
			AUC: 0.961

[26]	NB, C4.5, GA, and ACO	CNS	Accuracy: 85.00%
		Colon	Accuracy: 91.90%
		Leukemia_3C	Accuracy: 100%
		Leukemia_4C	Accuracy: 97.50%
		Leukemia	Accuracy: 100%
		Huntington disease	Accuracy: 100%
		DLBCL	Accuracy: 100%
		Lymphoma66 × 4026_3c	Accuracy: 100%
		Lymphoma	Accuracy: 93.21%
		Prostate	Accuracy: 95.00%
		Lung cancer	Accuracy: 100%
		Breast cancer	Accuracy: 97.18%
		Sarcoma	Accuracy: 75.36%
		Mycloma	Accuracy: 90.20%
		Ovarian	Accuracy: 98.80%

**Table 2:** Dataset description.

**Cancer name**	**Sample**	**Attributes**	**Class**
Lung cancer	253	12,533	2
Colon tumor	60	2000	2
Prostate cancer	103	341	2
Breast cancer	32	570	2
Leukemia cancer	72	7129	2

**Table 3:** Dimensionality after feature reduction and optimization.

**Dataset**	**After CFS**	**After IGWO**
Lung cancer (*D*_1_)	1250	320
Colon tumor (*D*_2_)	850	200
Prostate cancer (*D*_3_)	190	80
Breast cancer (*D*_4_)	2170	95
Leukemia cancer (*D*_5_)	2050	850

**Table 4:** Performance evaluation of various hybrid models in contrast to different datasets.

**Dataset**	**Methodology**	**ACC**	**PRE**	**MCC**	**F1-S**	**SEN**	**SPE**
*D*_1_	CFS + SVM	72.22	76.49	71.09	72.61	73.46	71.41
	CFS + MLP	70.61	74.68	75.68	76.67	74.07	72.53
	CFS + ELM	80.17	81.82	83.72	82.76	81.33	80.41
	CFS + AdaBoost	77.78	78.62	71.74	80.49	78.24	79.31
	CFS + DT	76.39	79.55	81.42	79.46	80.02	69.97
	CFS + KNN	79.89	76.12	74.12	75.22	74.11	72.13
	CFS-LR	71.56	73.98	68.56	74.56	73.11	71.12

*D*_2_	CFS + SVM	79.03	81.35	80.76	81.16	80.46	77.78
	CFS + MLP	82.26	86.49	81.21	85.33	84.66	79.17
	CFS + ELM	80.65	81.24	78.95	83.33	79.65	78.33
	CFS + AdaBoost	83.87	83.78	84.71	86.81	86.54	80.23
	CFS + DT	80.65	80.64	81.78	80.67	82.53	69.75
	CFS + KNN	78.45	71.67	67.89	65.11	71.12	70.12
	CFS-LR	78.34	79.45	69.89	71.45	73.23	74.11

*D*_3_	CFS + SVM	73.61	79.49	73.81	76.54	74.88	73.33
	CFS + MLP	70.83	72.22	70.27	71.23	70.65	71.43
	CFS + ELM	76.39	76.74	82.52	79.52	81.28	68.75
	CFS + AdaBoost	71.78	80.49	80.49	78.49	80.49	74.19
	CFS + DT	79.17	81.43	86.05	83.15	84.86	75.97
	CFS + KNN	72.22	71.34	76.23	74.56	78.67	70.11
	CFS + LR	72.11	74.34	78.23	61.23	70.12	73.13

*D*_4_	CFS + SVM	79.52	81.72	78.26	80.91	79.31	72.08
	CFS + MLP	77.11	82.61	77.55	78.12	76.51	76.47
	CFS + ELM	75.72	83.33	72.43	76.92	73.53	79.41
	CFS + AdaBoost	80.72	84.56	83.72	81.82	82.95	77.51
	CFS + DT	78.31	80.43	80.43	80.43	80.43	75.68
	CFS + KNN	65.13	71.34	72.11	78.56	73.23	72.45
	CFS + LR	77.22	76.45	78.44	72.33	72.23	76.14

*D*_5_	CFS + SVM	71.23	73.23	72.78	72.99	73.56	79.11
	CFS + MLP	78.34	80.11	82.13	81.45	82.89	85.78
	CFS + ELM	69.89	71.67	72.12	74.67	74.89	71.89
	CFS + AdaBoost	72.12	72.13	73.56	72.34	75.45	65.87
	CFS + DT	71.67	73.45	75.11	71.12	72.34	68.89
	CFS + KNN	69.23	70.45	71.34	72.13	77.67	72.74
	CFS + LR	68.89	78.56	75.23	74.23	71.89	73.34

**Table 5:** Performance of CFS-IGWO-DT.

**Dataset**	**ACC (%)**	**PRE (%)**	**SPE (%)**	**SEN (%)**	**F1-score (%)**	**MCC (%)**
Lung cancer	94.3	94.8	89.9	88.3	80.1	90.3
Colon tumor	92.2	93.1	91.2	94.8	82.2	80.1
Prostate cancer	91.3	92.2	92.3	92.1	83.1	81.2
Breast cancer	89.9	90.2	90.2	90.9	89.1	81.4
Leukemia cancer	88.1	86.1	86.2	86.2	89.3	83.3

**Table 6:** Performance of CFS-IGWO-LR.

**Dataset**	**ACC (%)**	**PRE (%)**	**SPE (%)**	**SEN (%)**	**F1-score (%)**	**MCC (%)**
Lung cancer	93.5	93.2	94.2	95.4	85.8	91.8
Colon tumor	92.9	89.6	90.3	93.2	89.7	90.7
Prostate cancer	90.5	88.8	91.3	90.4	87.4	89.5
Breast cancer	94.7	87.3	88.6	89.7	88.5	87.8
Leukemia cancer	89.9	90.4	83.6	88.4	89.1	85.3

**Table 7:** Performance of CFS-IGWO-MLP.

**Dataset**	**ACC (%)**	**PRE (%)**	**SPE (%)**	**SEN (%)**	**F1-score (%)**	**MCC (%)**
Lung cancer	95.7	89.9	90.9	88.9	89.9	90.2
Colon tumor	90.9	90.8	92.3	85.9	86.9	92.9
Prostate cancer	93.8	87.9	94.5	92.8	82.2	90.8
Breast cancer	94.5	86.9	90.4	95.9	84.4	94.6
Leukemia cancer	97.9	90.2	88.7	89.9	89.1	86.1

**Table 8:** Performance of CFS-IGWO-KNN.

**Dataset**	**ACC (%)**	**PRE (%)**	**SPE (%)**	**SEN (%)**	**F1-score (%)**	**MCC (%)**
Lung cancer	95.7	89.4	90.8	89.9	88.4	90.5
Colon tumor	94.3	91.3	93.4	82.5	87.3	89.3
Prostate cancer	90.7	86.5	92.5	89.7	85.3	88.2
Breast cancer	93.7	90.7	95.3	84.2	81.2	84.4
Leukemia cancer	90.6	88.4	91.1	86.2	82.2	85.3

**Table 9:** Performance of CFS-IGWO-SVM.

**Dataset**	**ACC (%)**	**PRE (%)**	**SPE (%)**	**SEN (%)**	**F1-score (%)**	**MCC (%)**
Lung cancer	97.8	93.6	94.5	94.9	89.9	88.9
Colon tumor	96.8	94.3	93.2	95.7	90.3	83.4
Prostate cancer	96.4	92.2	96.7	93.6	91.7	87.4
Breast cancer	95.6	91.5	90.6	92.8	91.8	90.1
Leukemia cancer	97.3	90.6	91.5	91.4	95.6	91.7

**Table 10:** Performance of CFS-IGWO-ELM.

**Dataset**	**ACC (%)**	**PRE (%)**	**SPE (%)**	**SEN (%)**	**F1-score (%)**	**MCC (%)**
Lung cancer	96.7	90.4	91.8	90.8	92.7	90.7
Colon tumor	95.7	91.5	92.3	97.6	91.8	89.9
Prostate cancer	94.6	94.5	95.4	95.4	90.7	88.6
Breast cancer	97.2	96.5	93.6	94.8	94.6	93.5
Leukemia cancer	94.3	93.4	95.6	96.5	95.7	92.9

**Table 11:** Performance of CFS-IGWO-AdaBoost.

**Dataset**	**ACC (%)**	**PRE (%)**	**SPE (%)**	**SEN (%)**	**F1-score (%)**	**MCC (%)**
Lung cancer	97.9	92.8	93.5	97.4	88.9	94.8
Colon tumor	96.8	95.6	96.4	96.8	89.6	92.5
Prostate cancer	94.6	97.5	95.6	95.6	90.4	93.7
Breast cancer	96.8	93.5	94.6	93.8	91.7	91.5
Leukemia cancer	97.5	94.3	93.8	94.2	93.8	89.9

**Table 12:** Output of ensemble classifiers for lung cancer.

**Ensemble classifier**	**Accuracy**	**Precision**	**Specificity**	**Sensitivity**	**F1-score**	**MCC**
Averaging	97.9	96.7	98.1	97.3	96.8	95.3
Voting	99.1	98.3	99.2	98.8	97.2	96.4

**Table 13:** Output of ensemble classifiers for colon tumor.

**Ensemble classifier**	**Accuracy**	**Precision**	**Specificity**	**Sensitivity**	**F1-score**	**MCC**
Averaging	95.6	96.7	97.9	96.4	97.9	95.3
Voting	97.8	98.7	99.1	98.9	98.7	97.4

**Table 14:** Output of ensemble classifiers for prostate cancer.

**Ensemble classifier**	**Accuracy**	**Precision**	**Specificity**	**Sensitivity**	**F1-score**	**MCC**
Averaging	97.8	98.4	96.4	98.9	95.4	97.7
Voting	99.3	97.6	98.4	99.2	97.5	98.6

**Table 15:** Output of ensemble classifiers for breast cancer.

**Ensemble classifier**	**Accuracy**	**Precision**	**Specificity**	**Sensitivity**	**F1-score**	**MCC**
Averaging	98.2	97.2	97.8	98.2	96.2	98.1
Voting	99.2	98.4	98.2	99.1	97.1	97.2

**Table 16:** Output of ensemble classifiers for leukemia cancer.

**Ensemble classifier**	**Accuracy**	**Precision**	**Specificity**	**Sensitivity**	**F1-score**	**MCC**
Averaging	97.1	97.4	99.1	97.3	96.4	97.8
Voting	98.7	99.1	98.2	98.6	97.5	98.2

**Table 17:** Accuracy comparison of different model with ensemble classifier.

**Models**	**Lung**	**Colon**	**Prostate**	**Breast**	**Leukemia**
CFS-IGWO-DT	94.3	92.2	91.3	89.9	88.1
CFS-IGWO-LR	93.5	92.9	90.5	94.7	89.9
CFS-IGWO-MLP	95.7	90.9	93.8	94.5	97.9
CFS-IGWO-KNN	95.8	94.3	90.7	93.7	90.6
CFS-IGWO-SVM	97.8	96.8	96.4	95.6	97.3
CFS-IGWO-ELM	96.7	95.7	94.6	97.2	94.3
CFS-IGWO-AdaBoost	97.9	96.8	94.6	96.8	97.5
AVERAGING	97.9	95.6	97.8	98.2	97.1
VOTING	99.1	97.8	99.3	99.2	98.7

**Table 18:** Performance analysis of the proposed model with existing literature.

**Ref**	**Lung**	**Colon**	**Prostate**	**Breast**	**Leukemia**
[13]	98.8%	83.8%	—	—	92.9%
[15]	98.8%	98.3%	—	—	98.9%
[17]	93.9%	97.8%	97.8%	89.5%	97.6%
[19]	94%	98%	—	86.5%	97%
[20]	97.5%	94.1%	—	50%	—
[21]	86.9%	—	—		87.5%
[22]	97.5%	93.5%	96.8%	86.6%	100%
[23]	91.7%	88.7%	82.9%		90.1%
Proposed	99.1%	97.8%	99.3%	99.2%	98.7%

## Data Availability

All data analyzed during this study are included in this article, and processed data is available on request from the corresponding author.
